# Safe and persistent growth-promoting effects of vosoritide in children with achondroplasia: 2-year results from an open-label, phase 3 extension study

**DOI:** 10.1038/s41436-021-01287-7

**Published:** 2021-08-02

**Authors:** Ravi Savarirayan, Louise Tofts, Melita Irving, William R. Wilcox, Carlos A. Bacino, Julie Hoover-Fong, Rosendo Ullot Font, Paul Harmatz, Frank Rutsch, Michael B. Bober, Lynda E. Polgreen, Ignacio Ginebreda, Klaus Mohnike, Joel Charrow, Daniel Hoernschemeyer, Keiichi Ozono, Yasemin Alanay, Paul Arundel, Yumiko Kotani, Natsuo Yasui, Klane K. White, Howard M. Saal, Antonio Leiva-Gea, Felipe Luna-González, Hiroshi Mochizuki, Donald Basel, Dania M. Porco, Kala Jayaram, Elena Fisheleva, Alice Huntsman-Labed, Jonathan R. S. Day

**Affiliations:** 1 Murdoch Children’s Research Institute, Royal Children’s Hospital, and University of Melbourne, Parkville, VIC Australia; 2 Kids Rehab, The Children’s Hospital at Westmead, Westmead, NSW Australia; 3 Guy’s and St. Thomas’ NHS Foundation Trust, Evelina Children’s Hospital, London, UK; 4 Department of Human Genetics, Emory University, Atlanta, GA USA; 5 Baylor College of Medicine, Houston, TX USA; 6 McKusick-Nathans Department of Genetic Medicine, Johns Hopkins University, Baltimore, MD USA; 7 Hospital Sant Joan de Déu, Barcelona, Spain; 8 UCSF Benioff Children’s Hospital Oakland, Oakland, CA USA; 9 Department of General Pediatrics, Muenster University Children’s Hospital, Muenster, Germany; 10 Nemours/Alfred I. du Pont Hospital for Children, Wilmington, DE USA; 11 Lundquist Institute for Biomedical Innovation at Harbor–UCLA Medical Center, Torrance, CA USA; 12 Hospital Universitario Quirón Dexeus, Barcelona, Spain; 13 Otto-von-Guericke-Universität, Magdeburg, Germany; 14 Ann and Robert H. Lurie Children’s Hospital of Chicago, Chicago, IL USA; 15 University of Missouri–Columbia, Columbia, MO USA; 16 Osaka University Hospital, Osaka, Japan; 17 Acibadem Mehmet Ali Aydinlar University, School of Medicine, Istanbul, Turkey; 18 Sheffield Children’s NHS Foundation Trust, Sheffield Children’s Hospital, Sheffield, UK; 19 Tokushima University Hospital, Tokushima, Japan; 20 Seattle Children’s Hospital, Seattle, WA USA; 21 Cincinnati Children’s Hospital Medical Center, University of Cincinnati College of Medicine, Cincinnati, OH USA; 22 Hospital Universitario Virgen de la Victoria, Málaga, Spain; 23 Saitama Children’s Hospital, Saitama, Japan; 24 Medical College of Wisconsin, Milwaukee, WI USA; 25 BioMarin Pharmaceutical Inc., Novato, CA USA; 26 BioMarin (U.K.) Limited, London, UK

## Abstract

**Purpose:**

Achondroplasia is caused by pathogenic variants in the fibroblast growth factor receptor 3 gene that lead to impaired endochondral ossification. Vosoritide, an analog of C-type natriuretic peptide, stimulates endochondral bone growth and is in development for the treatment of achondroplasia. This phase 3 extension study was conducted to document the efficacy and safety of continuous, daily vosoritide treatment in children with achondroplasia, and the two-year results are reported.

**Methods:**

After completing at least six months of a baseline observational growth study, and 52 weeks in a double-blind, placebo-controlled study, participants were eligible to continue treatment in an open-label extension study, where all participants received vosoritide at a dose of 15.0 μg/kg/day.

**Results:**

In children randomized to vosoritide, annualized growth velocity increased from 4.26 cm/year at baseline to 5.39 cm/year at 52 weeks and 5.52 cm/year at week 104. In children who crossed over from placebo to vosoritide in the extension study, annualized growth velocity increased from 3.81 cm/year at week 52 to 5.43 cm/year at week 104. No new adverse effects of vosoritide were detected.

**Conclusion:**

Vosoritide treatment has safe and persistent growth-promoting effects in children with achondroplasia treated daily for two years.

## INTRODUCTION

Achondroplasia is the most common form of disporportionate short stature in humans, caused by a common pathogenic variant in the fibroblast growth factor receptor 3 gene that confers a gain of function [1, 2]. People with achondroplasia experience significant medical and functional complications over their lifespan [2]. There are currently no approved precision therapies that target the underlying molecular etiology of this condition. Vosoritide, a modified C-type natriuretic peptide, stimulates endochondral ossification and is in clinical development to evaluate its safety and efficacy for the treatment of individuals with achondroplasia [3–5].

Studies in achondroplasia mouse models showed that subcutaneous administration of vosoritide increased long-bone and craniofacial growth [3, 4]. These data led to a growth study (to establish baseline growth over at least 6 months) and a phase 2, open-label study in children aged 5 to <14 years with achondroplasia [5]. The safety and efficacy data from this study supported further clinical development of vosoritide at a dose of 15.0-μg-per-kilogram-per-day in children with achondroplasia in pivotal, randomized controlled studies. This phase 3 study was a 52-week, randomized, double-blind, placebo-controlled design, and conducted in 121 children with achondroplasia aged 5 to <18 years. Eligible children were randomized 1:1 to treatment with vosoritide or an identical matching placebo [6]. The mean difference in annualized growth velocity between participants in the vosoritide group and placebo group was 1.57 cm per year in favor of vosoritide (95% CI: [1.22, 1.93], two-sided *p* value <0.0001) [6]. In total, 119 participants experienced at least one adverse event; 59 in the vosoritide group (98.3%), and 60 in the placebo group (98.4%) [6].

We report here an update from the phase 3 open-label extension study to document the efficacy and safety of up to two years of vosoritide treatment in children with achondroplasia.

## MATERIALS AND METHODS

Having completed at least six months of a baseline observational growth study (study 111-901; ClinicalTrials.gov number, NCT01603095), and 52 weeks in a double-blind, placebo-controlled, phase 3 study (study 111-301; EudraCT number, 2015-003836-11), children were then eligible to continue treatment in an open-label extension study (study 111-302; ClinicalTrials.gov number, NCT03424018).

In the placebo-controlled trial, children aged 5 to <18 years were randomized 1:1 to receive either vosoritide 15.0 µg/kg or placebo, for the duration of the 52-week treatment period administered by daily subcutaneous injections in their homes by trained caregivers. The dosing schedule was a single, daily subcutaneous injection given seven days a week, with regular injection site rotation. The clinical diagnosis of achondroplasia was confirmed by genetic testing. Children with radiographic evidence of closed growth plates, planned bone surgery, severe untreated sleep apnea, and other medical conditions or treatments known to impact growth were excluded. Written informed consent from a parent or legal guardian of each subject was obtained, and assent was obtained from the subject, if appropriate, prior to enrollment.

After completion of the placebo-controlled study, 119 children (*n* = 58 from the active arm and *n* = 61 from the placebo arm) were enrolled into the extension study, where all participants received vosoritide at a dose of 15.0 μg/kg/day. The data cut for this analysis occurred on 2 November 2020 when all ongoing participants had completed one year of follow up in the open-label extension study, which corresponds to two years on treatment for children originally randomized to vosoritide and one year on treatment for children who crossed over to vosoritide from placebo. Fifty-eight participants originally randomized to vosoritide continued vosoritide in the extension study. By week 104, *n* = 44 participants had standing height assessments available to determine six-month interval annualized growth velocity at the two year analysis time point. Sixty-one participants crossed over from placebo to vosoritide in the extension study and *n* = 47 had standing height assessments available to determine the six-month interval annualized growth velocity at the 2-year analysis time point. The cause of the missing data is principally due to disruptions to study visits due to the COVID-19 pandemic, where many site visits were replaced by virtual visits.

Descriptive summary plots for six-month interval annualized growth velocity are provided for the total of 121 children randomized to the placebo-controlled study using all available data from the baseline observational, randomized placebo-controlled and extension studies. A total of six six-month interval mean annualized growth velocity assessments were derived from standing height measurements commencing -52 weeks prior to randomization into the placebo-controlled study and concluding 104 weeks post randomization. The same summary plot was also produced where missing height data was imputed. Imputation for discontinued children was conducted as for the primary analyses of the randomized study by applying the baseline (pretreatment) annualized growth velocity to the last available height assessment. Linear interpolation was applied for the children who had missed the assessment but in whom an assessment at a later time point was available.

Standing height was converted to an age-appropriate and sex-appropriate *Z*-score by comparison with Centers for Disease Control and Prevention reference standards [7]. The upper-to-lower body segment ratio was calculated as the ratio between sitting height and standing height minus sitting height. Safety was evaluated by the incidence of adverse and serious adverse events.

## RESULTS

**Fig. 1:**
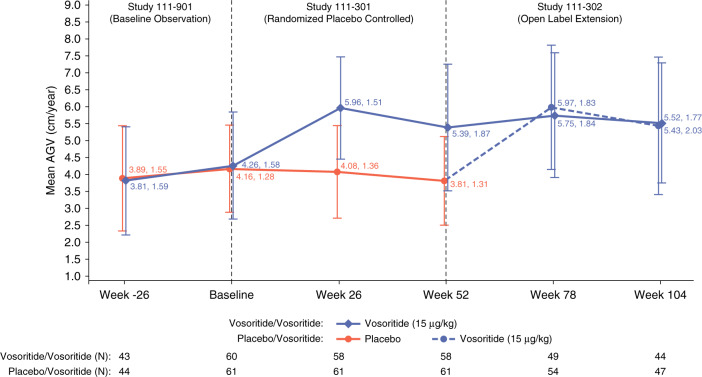
Line plot of mean annualized growth velocity shown in 6-month intervals starting in the baseline observation study and continuing through the randomized placebo-controlled study for 52 weeks and then into the extension study for a total of 104 weeks, displayed by treatment arm derived from observed data. Numbers at each time point reflect mean annualized growth velocity in cm/year and the standard deviation. Orange and dotted blue lines represent annualized growth velocity for participants randomized to the placebo study treatment arm and the solid blue lines represent annualized growth velocity for participants in the vosoritide study treatment arm. After 52 weeks and completion of the phase 3 study, 119 children were enrolled into the extension study, where all participants received vosoritide at a dose of 15 μg/kg/day. Fifty-eight participants originally randomized to vosoritide continued vosoritide in the extension study. By week 104, *n* = 44 participants had standing height assessments available to determine six-month interval annualized growth velocity at the two-year analysis time point. Sixty-one participants crossed over from placebo to vosoritide in the extension study and *n* = 47 had standing height assessments available to determine the six-month interval annualized growth velocity at the 2-year analysis time point. The cause of the missing data is disruptions to study visits due to the COVID-19 pandemic, where many site visits were replaced by virtual visits.

The annualized growth velocity was assessed overtime across the three studies over six-month intervals ([−12,−6 months], [−6,0 months], [0,6 months], [6,12 months], [12,18 months], and [18,24 months]) and included all children randomized into the placebo-controlled study (Fig. 1). The growth of participants during the baseline observational study was consistent between both placebo and vosoritide treatment groups. Baseline mean annualized growth velocity (SD) in children randomized to treatment with vosoritide was 3.81 (1.59) cm/year at –6 months and 4.26 (1.58) cm/year at baseline immediately before entering into the randomized study. In children randomized to treatment with placebo, the annualized growth velocity was 3.89 (1.55) cm/year at –6 months and 4.16 (1.28) cm/year at baseline.

In the placebo-controlled study, children randomized to treatment with vosoritide increased annualized growth velocity to 5.96 (1.51) cm/year at 26 weeks and 5.39 (1.87) cm/year at 52 weeks. In children randomized to placebo the annualized growth velocity was 4.08 (1.36) cm/year at 26 weeks and 3.81 (1.31) cm/year at 52 weeks.

The improvement in annualized growth velocity observed in children treated with vosoritide in the randomized placebo-controlled study was maintained in the open-label extension study with an annualized growth velocity of 5.75 (1.84) cm/year at week 78 and 5.52 (1.77) cm/year at week 104. Annualized growth velocity also increased to 5.97 (1.83) cm/year at week 78 and 5.43 (2.03) cm/year at week 104 in children who crossed over from placebo to vosoritide in the open-label extension study, resembling the initial treatment effect observed in the vosoritide arm at the start of treatment in the randomized study. Sensitivity analyses where all missing data were imputed were consistent with the summary plot on all observed data and are available in the supplementary information (Fig. S1). Spaghetti plots of standing height by age for individual participants are available in the supplementary information (Figs. S2–S5).

**Fig. 2:**
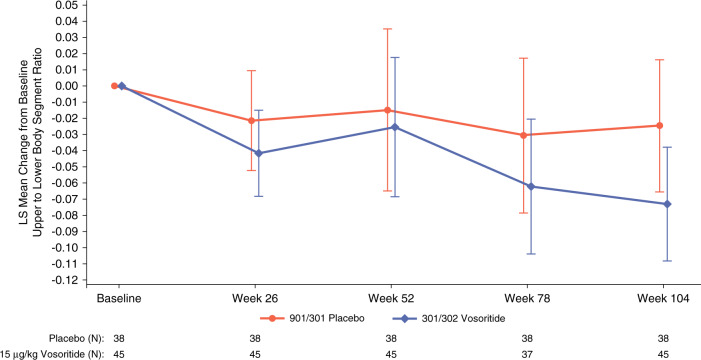
Line plot showing analysis of covariance (ANCOVA) LSmean change from baseline with 95% confidence intervals (CI) for upper-to-lower body segment ratio in 6-month intervals for a total of 24 months and displayed by treatment arm. Separate ANCOVA models provide the LSmean change from baseline at each time point for the participants who had completed the 2-year follow up. Orange lines represent change from baseline in upper-to-lower body segment ratio for participants randomized to the baseline observation study and the placebo study treatment arm and the solid blue lines represent change from baseline in upper-to-lower body segment ratio for participants in the vosoritide study treatment arm. Comparative analyses were conducted for all participants randomized to the active arm after two years on active treatment with an assessment at month 24 (*n* = 45) versus the participants in the placebo arm with two years of untreated follow up considering the one year placebo period and an additional year from the observational study prior to start of the randomized controlled study (*n* = 38). By directly comparing the treated group to the untreated group, comparative analyses at two years were performed using the same ANCOVA model, which adjusted for covariates, as prespecified for the primary and key secondary analyses of the randomized placebo-controlled study, the LSmean change from baseline in upper-to-lower body segment ratio (95% CI) was −0.05 (−0.09, −0.01) at week 104 representing a greater decrease in the body ratio in the vosoritide treated versus the untreated participants.

Comparative analyses were also conducted to assess the height gain for all participants randomized to the active arm after two years on active treatment with a height assessment at week 104 (*n* = 52) versus the participants in the placebo arm with two years of untreated follow up considering the placebo period and an additional year from the observational study prior to start of the randomized controlled study (*n* = 38). By directly comparing the treated group to the untreated group, the observed change in height was similar in the first year of treatment, 1.73 cm, as in the second year of treatment, 1.79 cm. The additional height gain over the two-year treatment period was 3.52 cm more than the untreated children. Comparative analyses at two years were performed using the same analysis of covariance (ANCOVA) model, which adjusted for covariates, as prespecified for the primary and key secondary analyses of the randomized placebo-controlled study, the LSmean difference (95% CI) was 3.34 cm (2.76, 3.93). Similarly, comparative analyses were also conducted to assess height *Z*-score and upper-to-lower body segment ratio. Using the same ANCOVA model, the difference in LSmean change in height *Z*-score (95% CI) was +0.44 (0.25, 0.63) at week 104 and the difference in LSmean change from baseline in upper-to-lower body segment ratio (95% CI) was −0.05 (−0.09, −0.01) at week 104 representing a greater decrease in the body ratio in the vosoritide treated versus the untreated participants (Fig. 2, Table S1).

No new adverse effects of vosoritide treatment at 15 μg/kg/day were detected with two years of continuous daily, subcutaneous treatment. Most adverse events were mild and no serious adverse events were attributed to vosoritide. The most common adverse event remains mild and transient injection site reactions.

Bone age was assessed with the Greulich and Pyle method using X-rays of the left hand and wrist. Bone age continued to progress normally in all children over the 104-week observation period. After one year of treatment, mean (SD) change from baseline in bone age considering assessments for both placebo and active arms was 1.10 years (0.89) for males (*n* = 46) and 1.01 years (0.94) for females (*n* = 41). The mean (SD) change from baseline in bone age after 2 years was 2.58 (1.33) years in males (*n* = 18) and 1.58 (1.42) years in females (*n* = 18).

## DISCUSSION

The effect of daily subcutaneous administration of vosoritide on growth as measured through annualized growth velocity and height *Z*-score was maintained for up to two years in children with achondroplasia aged 5 to 18 years, with an improvement in body proportions. There were no serious treatment-related adverse effects and vosoritide was well tolerated.

These data are consistent with those observed in the phase 2 and 3 clinical trials of vosoritide, where the increase in annualized growth velocity approached that of average-statured children of a similar age [5, 6]. They provide further, robust clinical evidence that vosoritide is an effective precision therapy for children aged 5 to 18 years with achondroplasia [5, 6]. No adverse effects on bone maturation have been observed in these trials. This, combined with the improvements in body segment proportionality, suggests that longer periods of treatment with vosoritide commenced at an earlier age might result in sustained enhancement of skeletal growth, with clinically and functionally beneficial consequences. Due to the inherent variability of growth and the lesser magnitude of the pubertal growth spurt in children with achondroplaisa, these long-term effects will only be known once these children reach final adult height [2].

The extension study reported here will also provide answers to whether vosoritide treatment will decrease the medical complications associated with achondroplasia, and improve functional outcomes. The study will collect data on other health measures, such as quality of life and incidence of expected complications, and these will be compared with registry data from untreated age-matched children with achondroplasia. This study will have the ability to detect if there are any long-term adverse effects of vosoritide therapy, and the effect of this therapy on pubertal growth acceleration in children with achondroplasia.

Other, ongoing clinical trials (ClinicalTrials.gov numbers NCT03583697, and NCT04554940) of vosoritide will investigate its safety and efficacy in children with achondroplasia aged 3 months to 60 months, and in infants at risk of requiring cervicomedullary decompression surgery [8]. These trials will provide further insights into the long-term treatment effects on skeletal growth, body proportions, and functionality, as well as how treatment might ameliorate the most significant medical complications in achondroplasia, specifically foramen magnum stenosis with brainstem compression and sudden death.

Overall, vosoritide treatment has safe and persistent growth-promoting effects in children with achondroplasia, and offers a precision therapy for patients impacted by this condition.

## Supplementary information

Supplementary Information

## Data Availability

The de-identified individual participant data that underlie the results reported in this article (including text, tables, figures, and appendices) will be made available together with the research protocol and data dictionaries, for noncommercial, academic purposes. Additional supporting documents may be available upon request. Investigators will be able to request access to these data and supporting documents via a website (www.BioMarin.com) beginning six months and ending two years after publication. Data associated with any ongoing development program will be made available within six months after approval of the relevant product. Requests must include a research proposal clarifying how the data will be used, including proposed analysis methodology. Research proposals will be evaluated relative to publicly available criteria at www.BioMarin.com to determine if access will be given, contingent upon execution of a data access agreement with BioMarin Pharmaceutical Inc.

## References

[1] Shiang R, Thompson LM, Zhu YZ, Church DM, Fielder TJ, Bocian M (1994). Mutations in the transmembrane domain of FGFR3 cause the most common genetic form of dwarfism, achondroplasia. Cell.

[2] Horton WA, Hall JG, Hecht JT (2007). Achondroplasia. Lancet.

[3] Lorget F, Kaci N, Peng J, Benoist-Lasselin C, Mugniery E, Oppeneer T (2012). Evaluation of the therapeutic potential of a CNP analog in a Fgfr3 mouse model recapitulating achondroplasia. Am J Hum Genet.

[4] Yasoda A, Komatsu Y, Chusho H, Miyazawa T, Ozasa A, Miura M (2004). Overexpression of CNP in chondrocytes rescues achondroplasia through a MAPK-dependent pathway. Nat Med.

[5] Savarirayan R, Irving M, Bacino CA, Bostwick B, Charrow J, Cormier-Daire V (2019). C-type natriuretic peptide analogue therapy in children with achondroplasia. N Engl J Med.

[6] Savarirayan R, Tofts L, Irving M, Wilcox W, Bacino CA, Hoover-Fong J (2020). Once-daily, subcutaneous vosoritide therapy in children with achondroplasia: a randomised, double-blind, phase 3, placebo-controlled, multicentre trial. Lancet..

[7] National Center for Health Statistics. Growth charts: United States. Atlanta: Centers for Disease Control and Prevention. https://www.cdc.gov/growthcharts/index.htm.

[8] Savarirayan R, Irving M, Maixner W (2021). Rationale, design, and methods of a randomized, controlled, open-label clinical trial with open-label extension to investigate the safety of vosoritide in infants, and young children with achondroplasia at risk of requiring cervicomedullary decompression surgery. Sci Prog.

