# Supplementary material for: Safe and persistent growth-promoting effects of vosoritide in children with achondroplasia: 2-year results from an open-label, phase 3 extension study
Source: Genet Med. 2021 Aug 2;23(12):2443–7. doi: 10.1038/s41436-021-01287-7 (PMC8327889; doi:10.1038/s41436-021-01287-7)
Supplement: Supplementary file 1 — Supplementary Information [file 41436_2021_1287_MOESM1_ESM.docx]

**Supplementary Information**

**Figure S1: Line plot of mean annualized growth velocity shown in 6-month intervals starting in the baseline observation study and continuing through the randomized placebo-controlled study for 52 weeks and then into the extension study for a total of 104 weeks, displayed by treatment arm derived from imputed data.**
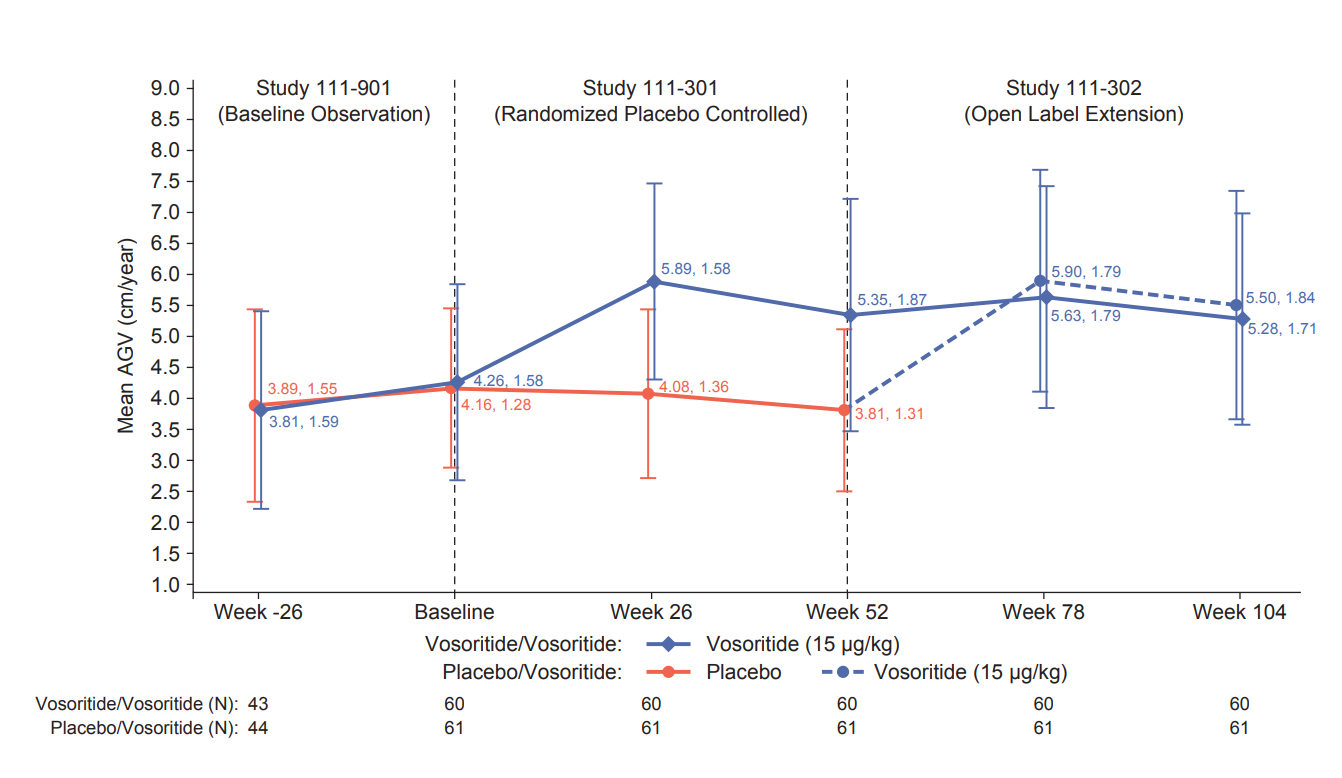


Line plot of mean annualized growth velocity shown in 6-month intervals starting in the baseline observation study and continuing through the randomized placebo-controlled study for 52 weeks and then into the extension study for a total of 104 weeks, displayed by treatment arm derived from imputed data. Numbers at each time point reflect mean annualized growth velocity in cm/year and standard deviation. Orange and dotted blue lines represent annualized growth velocity for participants randomized to the placebo study treatment arm and the solid blue lines represent annualized growth velocity for participants in the vosoritide study treatment arm. After 52 weeks and completion of the phase 3 study, 119 children were enrolled into the extension study, where all participants received vosoritide at a dose of 15 μg/kg/day. Sixty-one participants were originally randomized to vosoritide and 60 participants were originally randomized to placebo for one year and to later crossed-over to vosoritide in the extension study. Imputation for discontinued children was conducted as for the primary analyses of the randomized study by imputing the baseline annualized growth velocity to the last available height assessment. Linear interpolation was applied for the remaining subjects who had missed the assessment.

**Table S1: Completer analysis showing Analysis of Covariance for Upper to Lower Body Segment Ratio at Baseline and at 24 months**

| **Upper to Lower Body Segment Ratio** | **111-901/301 Placebo (N=38)** | **111-301/302 15 ug/kg BMN 111 (N=45)** |
| --- | --- | --- |
|  |  |  |
| **Baseline** |  |  |
| n | 38 | 45 |
| Mean (SD) | 2.00 (0.16) | 1.97 (0.20) |
| Median | 2.01 | 2.01 |
| 25th, 75th Percentile | 1.91, 2.12 | 1.89, 2.10 |
| Min, Max | 1.5, 2.4 | 1.3, 2.2 |
| **Month 24** |  |  |
| n | 38 | 45 |
| Mean (SD) | 1.95 (0.15) | 1.88 (0.21) |
| Median | 1.96 | 1.88 |
| 25th, 75th Percentile | 1.88, 2.07 | 1.79, 2.01 |
| Min, Max | 1.6, 2.2 | 1.3, 2.3 |
| **Change from baseline** |  |  |
| n | 38 | 45 |
| Mean (SD) | -0.05 (0.09) | -0.09 (0.11) |
| Median | -0.04 | -0.10 |
| 25th, 75th Percentile | -0.08, 0.01 | -0.17, -0.02 |
| Min, Max | -0.4, 0.1 | -0.3, 0.1 |
|  |  |  |
| **LS mean change from baseline (95% CI)** | -0.02 (-0.07, 0.02) | -0.07 (-0.11, -0.04) |
| **Difference in LS mean change from baseline (95% CI)^a^** |  | -0.05 (-0.09, -0.01) |

**Figure S2: Spaghetti Plot of Standing Height Over Time for all Male Participants randomized to Vosoritide followed by Vosoritide in the Extension Study**


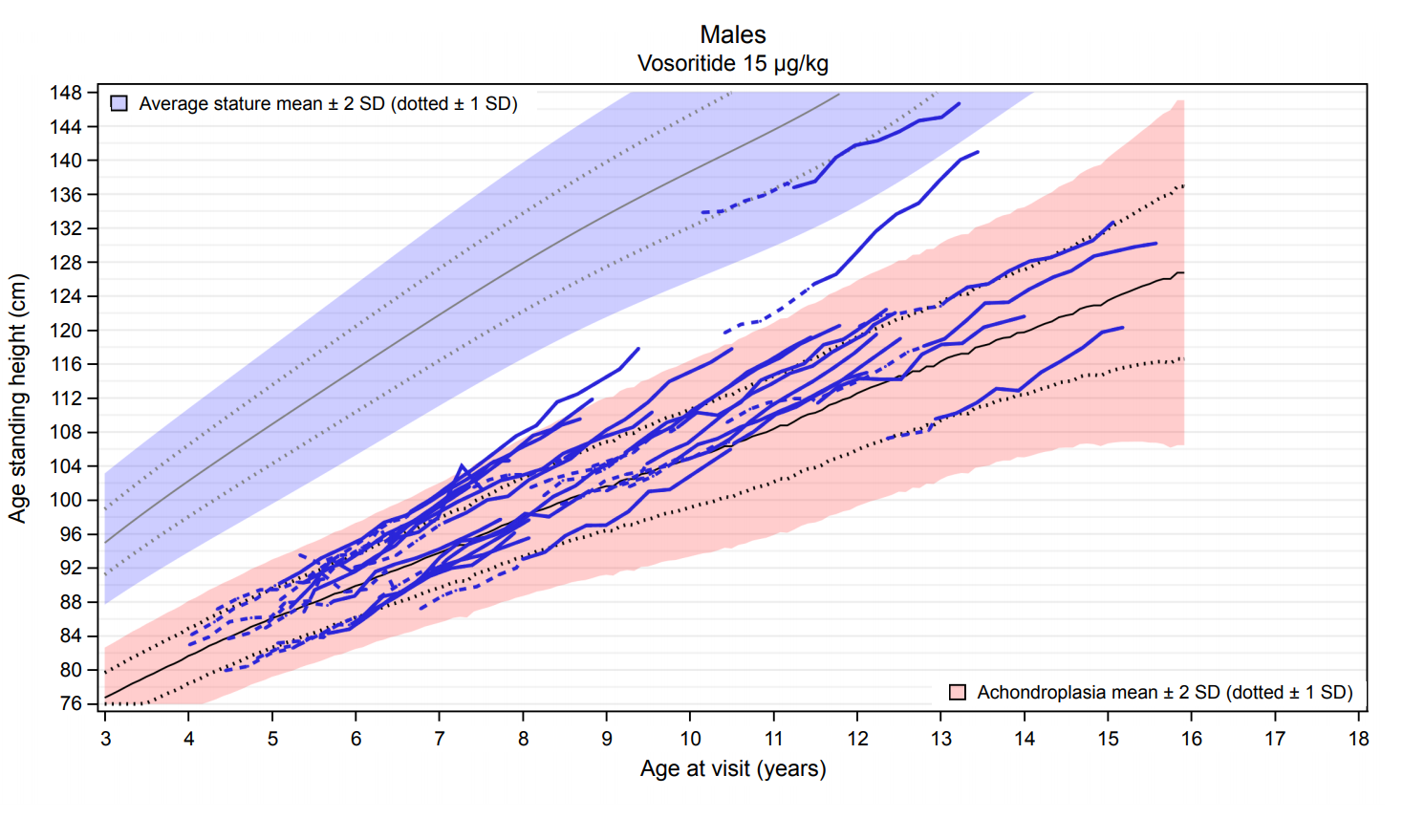


Individual dashed lines represent data collected before each participant received vosoritide and solid lines represent data collected when the participant was receiveing vosoritide. Reference ranges for average stature children are shown in the blue shaded area and were derived using age-sex specific reference data (means and SDs) per the Centers for Disease Control and Prevention. Reference ranges for achondroplasia are derived from Hoover-Fong et al. Am J Med Genet. 2017:173A:1226-1230.

**Figure S3: Spaghetti Plot of Standing Height Over Time for all Female Participants randomized to Vosoritide followed by Vosoritide in the Extension Study**


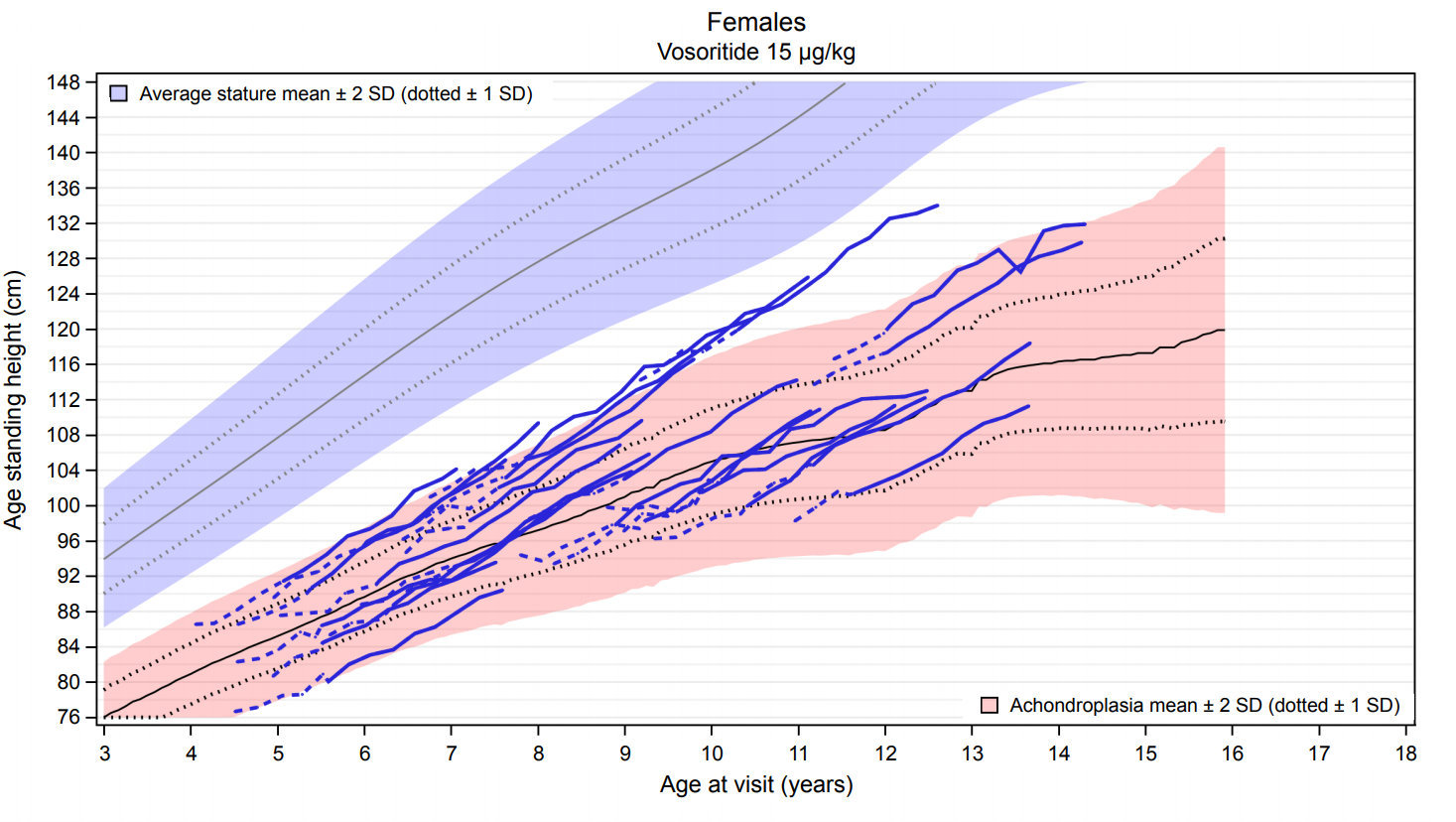


Individual dashed lines represent data collected before each participant received vosoritide and solid lines represent data collected when the participant was receiveing vosoritide. Reference ranges for average stature children are shown in the blue shaded area and were derived using age-sex specific reference data (means and SDs) per the Centers for Disease Control and Prevention. Reference ranges for achondroplasia are derived from Hoover-Fong et al. Am J Med Genet. 2017:173A:1226-1230.

**Figure S4: Spaghetti Plot of Standing Height Over Time for all Female Participants randomized to Placebo followed by Vosoritide in the Extension Study**


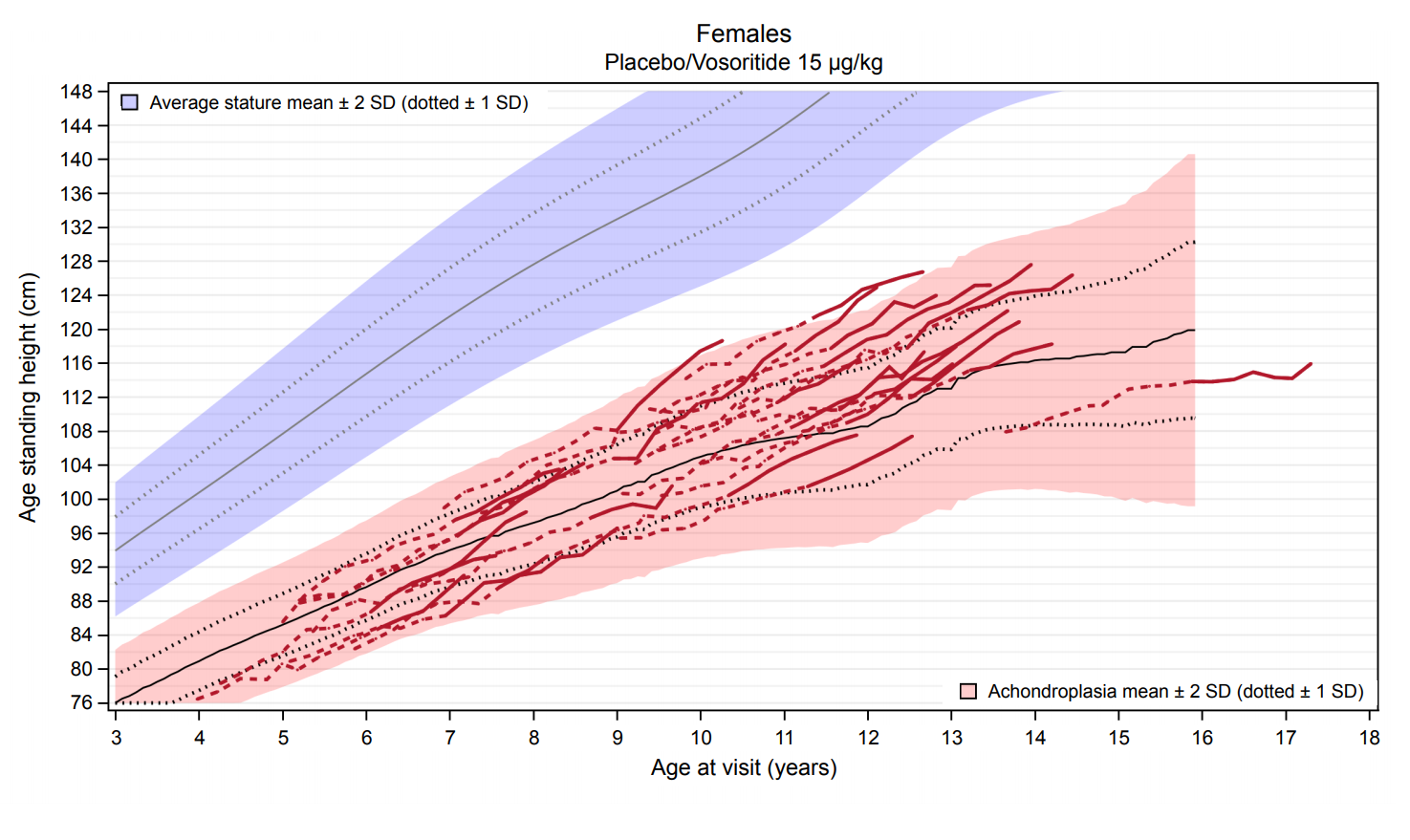


Individual dashed lines represent data collected before each participant received vosoritide and solid lines represent data collected when the participant was receiveing vosoritide. Reference ranges for average stature children are shown in the blue shaded area and were derived using age-sex specific reference data (means and SDs) per the Centers for Disease Control and Prevention. Reference ranges for achondroplasia are derived from Hoover-Fong et al. Am J Med Genet. 2017:173A:1226-1230.

**Figure S5: Spaghetti Plot of Standing Height Over Time for all Male Participants Randomized to Placebo followed by Vosoritide in the Extension Study**


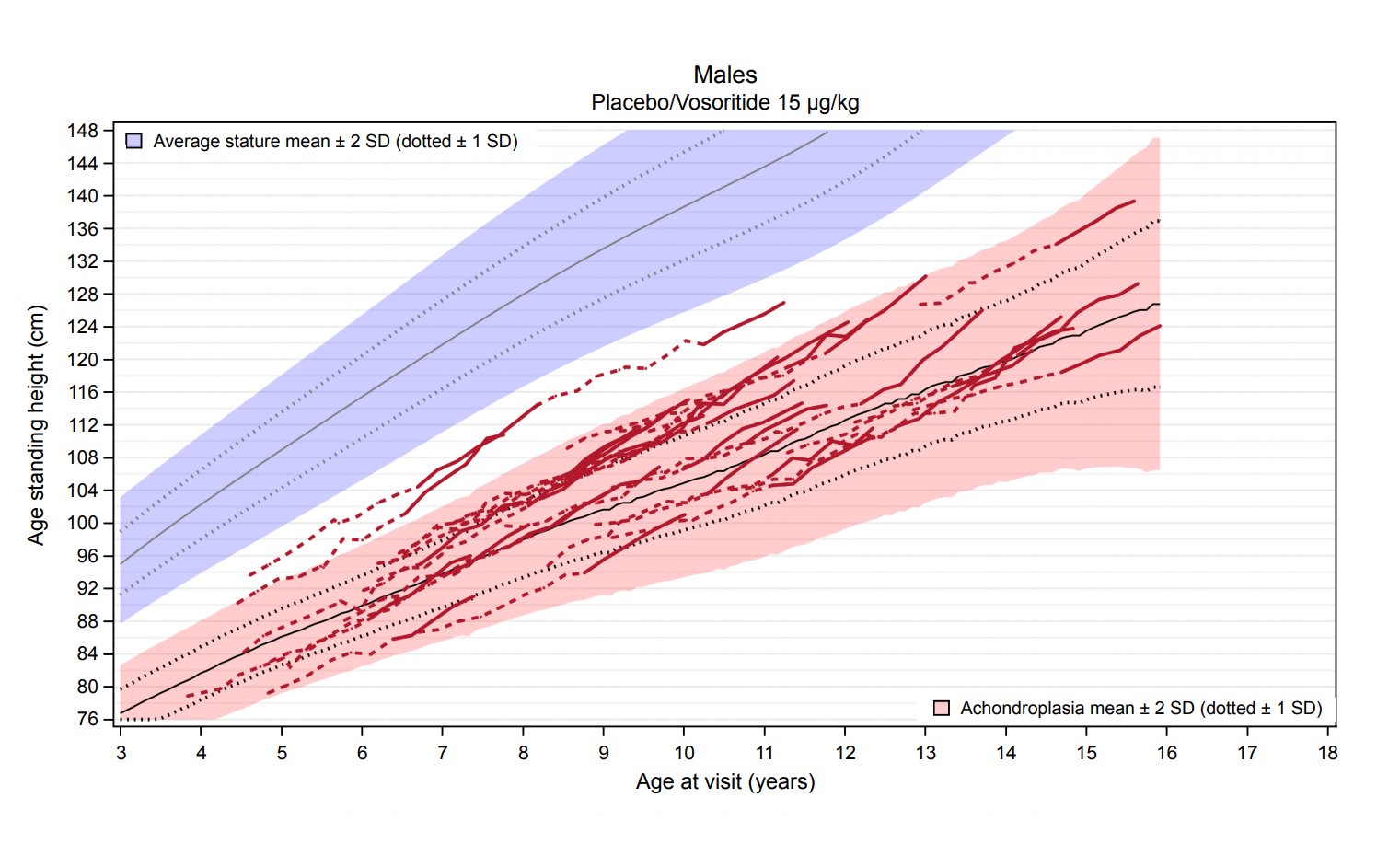


Individual dashed lines represent data collected before each participant received vosoritide and solid lines represent data collected when the participant was receiveing vosoritide. Reference ranges for average stature children are shown in the blue shaded area and were derived using age-sex specific reference data (means and SDs) per the Centers for Disease Control and Prevention. Reference ranges for achondroplasia are derived from Hoover-Fong et al. Am J Med Genet. 2017:173A:1226-1230.
